# Violencia obstétrica desde la percepción y vivencias de mujeres en labor de parto, Valledupar, Colombia

**DOI:** 10.15446/rsap.V26n6.115923

**Published:** 2024-11-01

**Authors:** Margareth Corzo-Contreras, Esther P. Polo-Payares, María C. Murgas-Quintero, Luz A. Hurtado-Luján, Belkis V. Cuesta-Morato

**Affiliations:** 1 MC: Enf. M. Sc. Salud Sexual y Reproductiva. Universidad Popular del Cesar. Valledupar, Colombia. margarethcorzo@unicesar.edu.co Universidad Popular del Cesar Salud Sexual y Reproductiva Universidad Popular del Cesar Valledupar Colombia; 2 EP: Enf. Esp. Materno Infantil. M. Sc. Estudios de Género Área Mujer y Desarrollo. Universidad de Cartagena, Cartagena, Colombia. epolop@unicartagena.edu.co Universidad de Cartagena Estudios de Género Área Mujer y Desarrollo Universidad de Cartagena Cartagena Colombia epolop@unicartagena.edu.co; 3 MM: Enf. Esp. Cuidado Neonatal. M. Sc. Salud Pública. Universidad Popular del Cesar. Valledupar, Colombia. mariamurgas@unicesar.edu.co Universidad Popular del Cesar Salud Pública Universidad Popular del Cesar Valledupar Colombia mariamurgas@unicesar.edu.co; 4 LH: Enf. Esp. Sistema de Calidad y Auditoría de Servicios de Salud. Universidad Popular del Cesar. Valledupar, Colombia. luzhurtado@unicesar.edu.co Universidad Popular del Cesar Sistema de Calidad y Auditoría de Servicios de Salud Universidad Popular del Cesar Valledupar Colombia luzhurtado@unicesar.edu.co; 5 BC: Enf: M. Sc. Salud Sexual y Reproductiva. Universidad Popular del Cesar. Valledupar, Colombia. belkiscuesta@unicesar.edu.co Universidad Popular del Cesar Salud Sexual y Reproductiva Universidad Popular del Cesar Valledupar Colombia belkiscuesta@unicesar.edu.co

**Keywords:** Atención hospitalaria, violencia obstétrica, distrés psicológico *(fuente: DeCS, BIREME)*, In-hospital care, obstetric violence, psychological distress *(source: MeSH, NLM)*

## Abstract

La violencia obstétrica ha sido definida desde legislaturas de Latinoamérica como todas las acciones u omisiones que el personal de salud ejerce sobre el cuerpo y los procesos reproductivos de las mujeres, consistentes en un trato deshumanizado, abuso de medicalización y patologización, que afecta su experiencia de parto.

**Objetivo:**

Visibilizar desde las voces de las mujeres su percepción de la vulneración de los derechos sexuales y reproductivos generados por la violencia obstétrica.

**Metodología:**

Estudio cualitativo, descriptivo exploratorio. Muestra intencional de 30 mujeres que cumplían los criterios de inclusión, usuarias de instituciones de salud de Valledupar, Colombia, entre 2020 y2021; se utilizó entrevista en profundidad y relatos orales, categorización teórica y emergente. Muestra con saturación, validación interna y externa por triangulación y consideración de aspectos éticos.

**Resultados:**

Las narrativas de las participantes dan cuenta de su percepción de las acciones que vulneran sus derechos, reflejada en expresiones que se interpretan a la luz de las recomendaciones de la Organización Mundial de la Salud (OMS), como el parto respetado, el abuso de medicalización y rutinas no recomendadas, que reflejan relaciones hegemónicas y no terapéuticas, silenciamiento e invasión de su corporalidad, así como riesgos para su salud y la de su hijo.

**Conclusión:**

La sumisión y falta de calidez experimentadas en un momento de vulnerabilidad de las mujeres, producen en su esfera psicológica la memoria de sus partos como unas experiencias negativas.

## Abstract (en)

Obstetric violence has been defined by Latin American legislatures as all actions or omissions that health personnel exercise on the body and reproductive processes of women, consisting of dehumanizing treatment, abuse of medicalization, pathologization and affecting their childbirth experience.

**Objective:**

To make visible from the women's voices their perception of the violation of sexual and reproductive rights generated by obstetric violence.

**Methodology:**

Qualitative, descriptive exploratory study. Intentional sample of 30 women who met the inclusion criteria, users of health institutions in Valledupar, Colombia, between 2020-2021; in-depth interview and oral accounts were used, theoretical and emergent categorization. Theoretical sample with saturation principle, internal and external validation by triangulation and consideration of ethical aspects.

**Results:**

The participants' narratives show their perception of actions that violate their rights reflected in expressions that are interpreted in the light of the recommendations of the World Health Organization (WHO), respected childbirth, as abuse of medicalization and non-recommended routines, in addition to reflecting hegemonic and non-therapeutic relationships, silencing and invasion of their corporeality and risks to their health and that of their child.

**Conclusion:**

Submission, lack of warmth, experienced in a moment of vulnerability of women, produces in their psychological sphere the memory of their deliveries as negative experiences.

La Organización Mundial de la Salud (OMS) promueve una atención digna, respetuosa, sin discriminación durante el proceso reproductivo de las mujeres, en concordancia con la meta del Tercer Objetivo de Desarrollo Sostenible, el cual consiste en "garantizar una vida sana para todos en todas las edades" 1. No obstante, distintos estudios en varios países evidencian las voces de las mujeres en torno a unas prácticas constitutivas de distintas formas de violencias, como verbal, psicológica o física, en relación con su experiencia en el embarazo, en particular en el parto, donde se plantea un panorama alarmante. Las mujeres son sometidas a tratos negligentes, humillaciones e incumplimiento de procesos institucionales como la obtención del consentimiento informado, además de la realización de procedimientos médicos sin previa información 2-4.

La historia ha demostrado que la atención del parto fue expropiada por la medicina moderna, eminentemente masculinizada desde el siglo XIX, lo cual implicó el desplazamiento de las parteras y la deslegitimación de las formas de llevar el proceso de parto y parir 5. Ello significó la vulneración de la mujer, el sometimiento a la administración de medicamentos oxitócicos para la aceleración del proceso natural del parto y la negación a la analgesia, lo que conlleva maltrato verbal, físico y psicológico 6, e incluso violencia simbólica, definida como la colaboración que ofrecen las personas que viven bajo una o varias formas de dominación con el orden social que las oprime, al punto que terminan considerando que las acciones médicas son naturales y de ayuda en el parto.

En este contexto, la violencia obstétrica es un fenómeno que conlleva acciones discriminatorias que se ejercen sobre la mujer en estado de gestación, durante el proceso de trabajo de parto, parto y puerperio, lo cual tiene un impacto negativo sobre esta experiencia. Además, acarrea la violación de los derechos humanos y reproductivos por parte de los profesionales de la salud, que actúan bajo la formación del habitus de poder, autoridad y desigualdad de género 7, oprimiendo los sentimientos de las mujeres y limitando sus expresiones de dolor de forma libre, sin ser burladas e infantilizadas con algún tipo sobrenombre o diminutivo 8. Estas acciones marcan a la mujer de manera significativa y derivan en afecciones emocionales.

En este sentido, distintos países han incorporado en sus legislaciones la violencia obstétrica como una forma de violencia de género, pero también como el reconocimiento de acciones que tipifican violencias y por tanto son punitivas 9.

Colombia, uno de los países que con el objetivo de reconocer y garantizar el derecho de la mujer durante el proceso reproductivo, el duelo gestacional y perinatal, respetando su libertad y conciencia, así como los derechos del recién nacido, aprobó la Ley 2244 de parto digno y humanizado 10.

Investigaciones realizadas en la Costa Caribe reportan cómo en las instituciones de salud públicas y privadas, el cuidado a la mujer durante el parto y posterior a este es negligente, distante y limitado a una relación de confianza que proporcione seguridad como base de la ética durante el proceso de atención, lo cual atropella la dignidad y la autonomía. Esta situación se acentúa debido a la precariedad de dotación, insumos e infraestructura de las instituciones de salud pública, donde las parturientas son víctimas de dichas condiciones y son estandarizadas, sin tener en cuentas sus creencias, condiciones biologías, riesgos y confort 8,11.

De la misma manera, la Corte Constitucional ha establecido una reciente línea jurisprudencial con respecto de la violencia obstétrica, particularmente, en las sentencias T-357 de 2021, SU-048 de 2022, T-198 de 2023 y T-576 de 2023. Por ello, que este artículo visibiliza la vulneración de los derechos sexuales y reproductivos de las mujeres atendidas institucionalmente en la ciudad de Valledupar.

## METODOLOGÍA

Estudio con enfoque cualitativo tipo descriptivo exploratorio, con el objetivo de visibilizar desde las voces de las mujeres su percepción de la vulneración de los derechos sexuales y reproductivos generada por la violencia obstétrica en instituciones de salud de la ciudad de Valledupar. Este abordaje permitió conocer la experiencia de las mujeres frente a dicho tipo de violencia, mediante los relatos y sus vivencias. El tipo descriptivo exploratorio permitió caracterizar la experiencia en el contexto 12. La población estuvo constituida por 30 mujeres que tuvieron parto hospitalario en un periodo no mayor a 6 meses, seleccionadas intencionalmente luego de cumplir los criterios de inclusión, que eran: tener más de 18 años, haber tenido parto institucional en los últimos 6 meses, no tener problemas de comprensión cognitiva y no haber experimentado depresión postparto.

Para esta investigación se asumió lo propuesto por Prado et al., quienes señalan que el muestreo teórico cualitativo consiste en obtener los conceptos relevantes que soportan las categorías empíricas. El principio de saturación se aplicó a las unidades de análisis, siendo estas cada entrevista, relato, narración e información que se haya obtenido de las participantes entrevistadas.

En una matriz cualitativa, se elaboró el sistema categorial, en el que se relacionan los objetivos de la investigación con las categorías predeterminadas mediante análisis teórico, según los aspectos incluyentes de la violencia obstétrica y las técnicas cualitativas de exploración a profundidad que contemplaron las subcategorías.

Para la recolección de la información se utilizó una ficha sociodemográfica, la cual se elaboró teniendo en cuenta las categorías más significativas que permitieron conocer e individualizar el perfil social y demográfico de las participantes. Además, se aplicó una entrevista en profundidad con preguntas orientadoras que responden a las categorías empíricas elaboradas por las investigadoras y que se sometió a validación facial y prueba piloto con ocho mujeres, a efectos de verificar su comprensión, en un espacio de naturalidad (Tabla 1).

**Tabla 1:** Matriz de categorías

Objetivo general	Objetivo específico	Categoría teórica	Categoría empírica	Técnica
Visibilizar desde las voces de las mujeres su percepción de la vulneración de los derechos sexuales y reproductivos generada por la violencia obstétrica	Caracterizar sociodemográfica ente a las usuarias del servicio de maternidad en instituciones de naturaleza pública o privada de la ciudad de Valledupar	Condiciones sociodemográficas	Factor socioeconómico: estrato, nivel de estudio Factor social: institución de salud de naturaleza pública o privada, nacionalidad, religión, estado civil. Condiciones biológicas: edad paridad, edad gestacional.	Ficha de perfil sociodemográfico (encuesta tipo censo)
	Develar en la narrativa de las mujeres, acciones de violencia obstétrica percibida por las usuarias en el servicio de maternidad	Violencia obstétrica en el contexto normativo: Ley de parto digno, respetado y humanizado, (Colombia 2022). Recomendaciones de la oms para los cuidados durante el parto, para una experiencia de parto positiva.	Percepción del parto como experiencia solitaria. Percepción de no ser escuchadas ni atendidas, sino regañadas. Percepción de solo recibir órdenes y tener que someterse sin opción. Narrativas que muestran uso de rutinas no recomendadas. Manipulación con la inducción de sentimientos de culpa para alinear el comportamiento.	Revisión fundamentada. Revisión sistematizada. Revisión bibliográfica. Entrevistas a profundidad con preguntas orientadoras. Relatos de situaciones en las que se identifiquen acciones de violencia.

Debido a la pandemia de COVID-19 y las restricciones sanitarias, así como las medidas de distanciamiento social, fue imposible llevar a cabo entrevistas presenciales. Por consiguiente, se optó por realizarlas de manera remota y estructurada, utilizando la modalidad telefónica, según la técnica Delphi 13 en la investigación cualitativa. Esta elección se basó en asegurar la seguridad y el bienestar tanto de las 30 participantes como del equipo investigador, de manera que se minimizara el riesgo de contagio. Esta estrategia no solo probó ser efectiva para cumplir con los objetivos del estudio, sino que destacó la capacidad de adaptación y resiliencia del equipo investigador.

Las participantes fueron seleccionadas mediante muestreo sistemático. De ellas, 15 fueron atendidas en una institución de salud de naturaleza pública y 15 en una institución privada. Además, para el reclutamiento se utilizó método de bola de nieve, en tanto que para participar en el estudio y grabar las conversaciones durante las llamadas, se solicitó el consentimiento informado. Antes de iniciar, se proporcionó una explicación detallada sobre el objetivo y los propósitos del estudio; se dejó claro que las participantes podían retirarse voluntariamente en cualquier momento sin tener por ello consecuencias negativas.

Las entrevistas fueron concertadas de acuerdo con la disponibilidad de las participantes, evitando así obstáculos que se pudieran presentar. Algunas de las entrevistas se realizaron en dos sesiones, debido a la profundidad de los relatos. Estos relatos fueron transcritos, interpretados y verificados por las investigadoras para asegurar su fidelidad y, con el fin de que las participantes se sintieran representadas en los escritos, estos fueron revisados nuevamente. La lectura de sus respuestas, el análisis por parte de los autores y la confirmación de las participantes fueron fundamentales en este proceso, dado que permitió recolectar y reconocer información sobre sus pensamientos y sentimientos.

### Análisis de datos

En el procesamiento y el análisis de la información se establecieron códigos para identificar elementos de las participantes. De esta manera, en relación con las categorías empíricas del factor social de la institución de salud pública o privada MIPV (multigestante institución privada), PIPV (primigestante institución privada), MIPB (multigestante institución pública) y PIPB (primigestante institución pública), los números hacen referencia a la secuencia en que las participantes fueron entrevistadas. Se utilizaron pseudónimos, como se puede observar al final de cada relato (Tabla 2).

**Tabla 2:** Perfil sociodemográfico

Código	Pseudónimo	Factor socioeconómico			Factos social			Condiciones biológicas		
		Nivel de estudio	Estrato	Estado civil	Religión	Nacionalidad	Institución de salud	Edad	Número de hijos	Semana de gestación
PIPV1	Gloria	Técnico	1	Soltera	Cristiana	Colombia	Privada	20	1	39
MIPB1	Dania	Primaria	1	Soltera	Ninguna	Venezuela	Publica	24	3	37
PIPV2	Pierina	Bachiller	1	Unión libre	Católica	Colombia	Privada	24	1	40
MIPB2	Sol	Técnica	2	Unión libre	Católica	Colombia	Publica	40	2	38
PIPV3	Carmen	Técnico	2	Unión libre	cristiana	Colombia	Privada	32	1	38
PIPB3	Johana	Bachiller	2	Unión libre	Ninguna	Venezuela	Publica	24	1	42
PIPV4	Juana	Técnico	2	Unión libre	cristiana	Colombia	Privada	33	1	39
PIPB4	Yennys	Bachiller	1	Soltera	Ninguna	Colombia	Publica	19	1	38,5
MIPV5	Karina	Universitaria	2	Casada	Católica	Colombia	Privada	29	2	36,3
MIPB5	Patricia	Bachiller	2	Unión libre	Ninguna	Venezuela	Publica	33	4	41
MIPV6	Tatiana	Bachiller	2	Unión libre	Católica	Colombia	Privada	33	3	38
MIPB6	Andrea	Bachiller	1	Unión libre	Ninguna	Venezuela	Publica	19	2	39
MIPV7	Mónica	Bachiller	2	Unión libre	Ninguna	Colombia	Privada	27	4	40
MIPB7	Laura	Bachiller	2	Soltera	Católica	Venezuela	Publica	24	4	39,4
MIPV8	Margarita	Universitaria	2	Casada	Católica	Colombia	Privada	34	3	37,5
PIPB8	Ana	Bachiller	2	Soltera	Cristiana	Venezuela	Publica	18	1	37,5
MIPV9	Yaniris	Técnico	1	Unión libre	Ninguna	Colombia	Privada	33	2	38
MIPB9	Gladis	Bachiller	2	Unión libre	Cristiana	Venezuela	Publica	23	6	26
PIPV10	Angela	Técnico	3	Casada	Ninguna	Colombia	Privada	38	1	38
MIPB10	Paola	Universitaria	2	Unión libre	Ninguna	Venezuela	Publica	27	3	40
PIPV11	Mercedes	Técnico	3	Unión libre	Católica	Colombia	Privada	30	1	38
MIPB11	Lorena	Bachiller	1	Unión libre	Ninguna	Venezuela	Publica	29	4	39,5
MIPV12	Luciana	Universitaria	3	Casada	Católica	Colombia	Privada	34	2	36
MIPB12	Lina	Bachiller	1	Unión libre	Ninguna	Venezuela	Publica	25	3	38,5
PIPV13	Elen	Bachiller	1	Unión Libre	Católica	Colombia	Privada	18	1	38
MIPB13	Liceth	Bachiller	1	Unión libre	Cristiana	Venezuela	Publica	37	6	39
PIPV14	María	Técnico	3	Unión libre	Cristiana	Colombia	Privada	31	1	38
PIPB14	Yurian	Bachiller	1	Soltera	Cristiana	Venezuela	Publica	18	1	37
PIPV15	Zulma	Universitaria	1	Unión libre	Católica	Colombia	Privada	24	1	39,1
MIPB15	Claudia	Primaria	1	Unión libre	Católica	Venezuela	Publica	25	4	34

La información se organizó en matrices de análisis cualitativo, las cuales sirvieron para documentar las categorías y las subcategorías. Esta información, luego de ser codificada, se sometió a proceso de triangulación entre participantes, autores de otros referentes externos y narrativas de otras participantes, así como el pronunciamiento y los lineamientos de autoridades en salud como la OMS y el Ministerio de Protección Social de Colombia, lo cual corresponde al criterio de transferibilidad. Dado que se exploraron las situaciones específicas vividas por mujeres, se tuvo en cuenta su significado en contexto como otro criterio de rigor.

En el análisis se procuró la correspondencia entre los relatos de la experiencia durante el trabajo de parto, el parto y el puerperio, según lo expresado por las participantes, con el fin de verificar su coherencia, según lo presupuestado por Sampieri 12. Se llevó a cabo un análisis de triangulación, lo que permite investigadoras señalar otros resultados que han mostrado violencia obstétrica, así como otros que se han obtenido con el uso de metodologías cualitativas que, en un futuro, también podrían llegar a resultados similares.

### Criterios de rigor

La información recolectada se digito fidedignamente, siguiendo los criterios de rigor planteados por Lincoln et al.: fidedignidad y reciprocidad, de acuerdo con los cuales se sometió a verificación con las participantes, la correcta interpretación de sus términos y metáforas. En el texto, estos preservan su originalidad, minimizando los sesgos, las reflexiones y las concepciones de las investigadoras durante la interpretación de los datos, con el propósito de mantener la credibilidad y la autenticidad.

### Consideraciones éticas

La investigación contó con aval de una universidad acreditada con alta calidad de Colombia. Se categorizó como una investigación de riesgo mínimo, según lo contemplado en la Resolución 08430 de 1993 de investigación en humanos.

Las investigadoras estaban entrenadas de acuerdo con las recomendaciones de la OMS, para hacer contención de emociones y aplicar las sugerencias de seguridad en investigaciones sobre violencia contra la mujer. Es menester aclarar que durante la recolección de la información (entrevistas) no se presentaron episodios de catarsis emocional en las participantes.

Se tuvieron en cuenta aspectos éticos relacionados con la autonomía, la confidencialidad y el consentimiento de los sujetos participantes en la investigación y el permiso de uso de audios. Asimismo, se aclaró que la participación era voluntaria y anónima y que los resultados serían utilizados con fines ajenos al ámbito científico.

## RESULTADOS

El estudio estuvo conformado por 30 mujeres que vivieron la experiencia del parto en instituciones de salud de naturaleza pública y privada de la ciudad de Valledupar, Cesar. La información en torno al perfil sociodemográfico se presenta en la Tabla 3.

**Tabla 3:** Perfil sociodemográfico

Categoría	Datos
Edad	Entre 18 y 25 años: 12 mujeres.
	Entre 26 y 35 años: 15 mujeres.
	Entre 36 y 40 años: 3 mujeres.
Nivel de estudio	5: Grado universitario.
	8: Estudios técnicos.
	15: Secundaria completa.
	2: Primaria completa.
Estrato	Estrato I: 12
	Estrato II: 13
	Estrato III: 5
Estado civil	Solteras: 6 mujeres
	Casadas: 4 mujeres
	Unión Libre: 20 mujeres
Religión	Católicas: 12 mujeres
	Evangélicas: 7 mujeres
	No profesan ninguna religión: 11 mujeres
Nacionalidad	Colombianas: 17 mujeres
	Venezolanas: 13 mujeres
Antecedentes obstétricos	13: Primíparas
	17: Multíparas
Institución	Pública: 15 mujeres
	Privada: 15 mujeres

### Percepción del parto como experiencia solitaria

En los siguientes relatos podemos visualizar cómo las mujeres percibieron su parto como una experiencia desagradable, debido a sentirse solas: "Esta experiencia en el hospital fue desagradable, no le dan a uno buen trato, ni los médicos, ni las enfermeras; además que uno se la pasa es solo" [Yennys (PIPB4)].

"Desde que ingresé yo estuve sola hasta que me pasaron pá piso, ahí uno si se siente tristongo porque, aja uno quiere estar es acompañado que le estén dando apoyo a uno en ese momento. [Tatiana (MIPV6)]."Estaba completamente aislada de familiares sin quien le diera a uno esa fortaleza que necesita en ese momento" [Luciana (MIPV12)].

#### Percepción de no ser escuchadas ni atendidas, sino regañadas

MIPV12 y MIPB1 narran el drama que tuvieron que experimentar como negligencia de los profesionales de la salud: "Llamé otra vez a la muchacha a la enfermera porque ya yo sentía que el bebé se venía, comencé a gritar que me ayudarán, que me ayudaran que el bebé ya venía en camino entonces fue cuando salieron corriendo me llevaron caminando a sala de parto caminando que me agarrara el bebé que por que no había avisado dijo una doctora que me lo tuviera me tocó sostenerle la cabeza al bebé con la mano mientras íbamos" [Luciana (MIPV12)].

#### Percepción de solo recibir órdenes y tener que someterse sin opción

Esto se denota en las siguientes narraciones de las mujeres al momento de su expulsivo, donde la posición del parto parece ser una imposición: "Bueno yo no elegí, creo que no estaba en posición de elegir, simplemente ellos ahí estaba la camilla, me montaron, me explicaron dónde deberían ir las piernas, de dónde tenía que agarrarme, qué posición debía tener para pujar, o sea, la posición ideal para parir y que todo fuera lo más rápido, eso me lo iba explicando el médico" [Juana (PIPV4)]. "Nunca me preguntaron, ni me dieron opción de nada, simplemente me dijeron súbase a la camilla y esperé ahí" [Mercedes (PIPVH)].

#### Narrativas que muestran uso de rutinas no recomendadas

Las participantes refirieron haber sido sometidas a prácticas orientadas a la aceleración de su proceso fisiológico (parto), como ruptura prematura de membranas de manera artificial, maniobra de Kristeller y episiotomía, sin información y autorización: "Una doctora dijo que ya estaba en 6 y que me iba a romper las membranas para que avanzara, y una enfermera que estaba ahí la ayudó" [Yennys (PIPB4)]. "Varios doctores me hicieron el tacto me decían en cuantos centímetros iba y la cuestión del trabajo de parto me colocaron un medicamento, me rompieron las membranas, estaba en 5 de dilatación cuando la doctora me la rompió, me dijo que agilizaba más rápido el trabajo de parto" [Lina (MIPB12)]. "Entonces el momento él agarra el bisturí supongo y me cortó hacia abajo ¡Uish!, realmente no fue dolor fue como ardor, por que como el dolor es tan fuerte que eso es mínimo, uno no lo siente, pero si sentí el ardor de que me puso algo y que cortó, sentí el ardor yo grité, porque fue algo que yo no me esperaba, pero él me decía tranquila, tranquila y yo en lo que pude traté de tranquilizarme" [Gloria (PIPV1)]. "Yo sentí como una molestia, y yo ¿qué es eso? me estaban cosiendo, no sentí cuando me picaron, pero si cuando me estaban cosiendo" Qohana (PIPB3].

Otra de las intervenciones realizadas de forma rutinaria para acelerar el proceso de parto que se identifica en las narraciones de las participantes es la medicalización, la cual lleva consigo la sujeción de la mujer a un dispositivo tecnológico (bomba de infusión). Esta alternativa reduce la movilidad y la independencia de la parturienta.

De acuerdo con las narraciones de las participantes, los tactos vaginales fueron realizados por diferentes profesionales, incluso en numerosas ocasiones: "Me pusieron la pitocina para que me diera dolor porque todavía no me había empezado a dar dolor, no me pidieron permiso, si me dijeron que me iban a poner para que me diera dolor, para que me ayudara" [Tatiana (MIPV6)]. "Me pusieron la oxitócica, ahí con un cosito ahí que medía el tiempo de cuanto, cuanto no sé y me daban las contracciones, ellos la colocaron y ya, Sí, pero igual yo ya lo sabía que era para contracción que fuera más rápido" [Yaniris (MIPB9)]. "Me hicieron como 6 tactos, me lo hicieron como 3 personas diferentes era el médico y los estudiantes de medicina" [Patricia (MIPB5)].

#### Manipulación con la inducción de sentimientos de culpa para alinear el comportamiento

Las participantes refirieron haber recibido palabras que las responsabilizaban de la posible muerte de sus bebés: 'Ajá me dijeron que no pujara hacia abajo por qué vas a matar la niña, la vas a ahogar porque yo pujaba era así hacía abajo, me decían no pujes hacia abajo que tienes bebé ahí me decía el doctor que me estaba atendiendo el parto" [Diana (MIPB1)]. "Me decían que me apurara que mantuviera el pujo que a mí se me podía ahogar, que ya eran cinco niños y que sabía cómo tenía que hacerlo, eso era lo que ellos me decían" [Laura (MIPB7)].

## DISCUSIÓN

El presente estudio cualitativo explora las experiencias de mujeres que han sido víctimas de violencia obstétrica, identificando y analizando las percepciones y sentimientos asociados con este fenómeno. A través de entrevistas detalladas, emergieron cinco categorías clave que delinean la naturaleza de estas experiencias: la percepción del parto como una experiencia solitaria; la percepción de no ser escuchadas ni atendidas, sino regañadas; la percepción de recibir solo órdenes y tener que someterse sin opción; el uso de rutinas no recomendadas; y la manipulación con la inducción de sentimientos de culpa para alinear el comportamiento.

El sentimiento de soledad genera la idea de estar desprotegidas; la institucionalización del parto trajo consigo que la mujeres dejaran de vivir el nacimiento de sus hijos como un acontecimiento en la vida familiar en sus domicilios, con participación y apoyo de otros miembros de su núcleo familiar. Por ello, todas las iniciativas tanto gubernamentales como de guías de práctica clínica intentan rescatar esto a través de permitir el acompañamiento de un familiar escogido por la mujer, explícito en la Ley de Parto Seguro y Humanizado en Colombia 8.

A pesar de que los profesionales sanitarios tienen la responsabilidad de brindar cuidados con calidad, apuntando al logro de la excelencia en la atención humanizada, en los relatos de las participantes se reflejan experiencias negativas con el personal sanitario, en el sentido de omitir recomendaciones de la OMS y de la Política de Salud Sexual y Reproductiva; imponer protocolos institucionales; y someter a la mujer a medicalización y negligencia.

Las prácticas rutinarias que no recomendadas pueden generar desenlaces desfavorables que comprometan la vida y la integridad de las madres y los bebés. En las narrativas de las participantes se encontró el uso de la maniobra de Kristeller, la amniotomía mecánica y el uso de oxitocina para acelerar el trabajo de parto.

Esta investigación evidenció la inducción del sentimiento de culpa como un mecanismo para mantener el control y conseguir la cooperación de la parturienta, consistente con lo encontrado por Annborn y Rafnar 15, quienes reportan el uso del mismo mecanismo.

Centrar en las mujeres en trabajo de parto la responsabilidad del desenlace negativo, pudiese generar en el personal clínico un mecanismo de control aparentemente efectivo, pero en ellas ocasiona un distrés psicológico que puede repercutir en un efecto contrario a lo esperado.

En este estudio se demostró que las participantes percibieron formas diversas de violencia como la verbal, la psicológica y la física en el uso de procedimientos rutinarios ya no recomendados, así como trato deshumanizado. En este sentido, la praxis más frecuente fue el mantenerlas aisladas de sus familiares o de un acompañante de confianza, así como ser criticadas por expresar sus sentimientos, acelerar el parto por medio de la medicalización, repetir constantemente el tacto vaginal, realizar episiotomías, desatención, todo lo cual les hace sentir temor, incertidumbre, vulnerabilidad e inseguridad.

El periodo de parto es una experiencia importante que marca a la mujer de manera significativa. Aunque puede repercutir positivamente en lo psicológico, también puede generar afectaciones emocionales como la culpa, debido a sentimientos de responsabilidad frente a sucesos acaecidos durante el proceso de parto, si los resultados durante este son negativos. Lo anterior aumenta el riesgo de depresión posparto, lo cual afecta al recién nacido y al entorno familiar ♦
